# Advances in the biological effects of terahertz wave radiation

**DOI:** 10.1186/s40779-014-0026-x

**Published:** 2014-12-02

**Authors:** Li Zhao, Yan-Hui Hao, Rui-Yun Peng

**Affiliations:** Department of Experimental Pathology, Beijing Institute of Radiation Medicine, Beijing, 100850 China

**Keywords:** Terahertz, Radiation, Biological effects, Progress

## Abstract

The terahertz (THz) band lies between microwave and infrared rays in wavelength and consists of non-ionizing radiation. Both domestic and foreign research institutions, including the army, have attached considerable importance to the research and development of THz technology because this radiation exhibits both photon-like and electron-like properties, which grant it considerable application value and potential. With the rapid development of THz technology and related applications, studies of the biological effects of THz radiation have become a major focus in the field of life sciences. Research in this field has only just begun, both at home and abroad. In this paper, research progress with respect to THz radiation, including its biological effects, mechanisms and methods of protection, will be reviewed.

## Introduction

Essentially, THz is an electromagnetic wave with frequencies ranging from 0.3 THz to 3 THz and wavelengths ranging from 0.1 mm to 1 mm. THz waves were formally named in the late 1980s, before which time they were collectively referred to as far-infrared rays. Because of the limited availability of effective THz-generating sources and THz-sensitive detectors, there has been very little research into this band; therefore, this band is also called the THz Gap. The term THz Gap is applicable in the following two respects: First, the THz band is located between millimeter waves and infrared rays, both of which bands have been more extensively investigated, thus forming a relatively undeveloped “blank.” Second, studies of the long-wavelength regime, of which the THz band is a part, rely predominantly on electronics, whereas studies of the short-wavelength regime rely predominantly on photonics, resulting in a “gap” between these two research fields.

Compared with other bands, the THz band offers the following unique advantages: First, THz pulse widths on the subpicosecond scale allow for the facile analysis of various materials (such as liquids, semiconductors, superconductors, biological samples, etc.) through the technology of time-resolved transient spectroscopy. Second, a THz pulse source must produce only a few cycles of oscillation, and the frequency range of each single pulse spans from the range of GHz (giga-Hz) to 10 THz. This feature of THz sources is beneficial for the analysis of the spectral properties of a wide range of sample materials. Third, THz radiation is highly coherent, including strong temporal coherence and strong spatial coherence. This property assists in the accurate acquisition of the refractive indices and absorption coefficients of samples. Fourth, THz photons possess very low energies; therefore, THz radiation does not cause tissue damage or harmful ionization and is thus highly suitable for biopsy. Fifth, THz radiation offers strong penetration power for non-polar substances and can be used for detection. Sixth, THz waves are strongly absorbed by polar molecules, meaning that THz radiation can be used to study material compositions by analyzing the corresponding spectroscopic characterizations of the materials and can be used in product quality control.

In recent years, research and technology in the field of THz science has undergone tremendous development. In addition to THz radiation sources, remarkable developments have also been made in traditional THz detection, THz spectroscopy, THz imaging studies, THz radar, THz remote sensing, THz communication, THz measurement, and THz applications for non-damaging methods of detection as well as THz technology for use in material characterization and environmental monitoring. Research has been conducted in the petrochemical, aerospace, and biomedical industries as well as by the military and agencies concerned with national defense and national security, among others. Naturally, this extensive research program has aroused worldwide concern. With the rapid development of THz science and technology, exploration of the biological effects of THz radiation has become a major new effort in the field of life sciences. At present, research in this field, both at home and abroad, is still in its initial stages.

In this paper, research progress with respect to THz radiation, including its biological effects, mechanisms and methods of protection, will be reviewed.

## Biological effects of THz radiation

At present, studies of the biological effects of THz radiation are largely supported by “THz-BRIDGE,” an international cooperative project with the objective of studying the mechanisms involved in the interaction of THz radiation with cells and biological molecules to estimate the potential genotoxicity of THz radiation [1]. Meanwhile, the project is also committed to identifying the potential target organs, cells or sites of THz radiation, mostly focusing on lymphocytes, epidermal cells and cell membranes [2].

Similar to other bands of the electromagnetic spectrum, the biological effects of THz waves can be divided into thermal effects and non-thermal effects. Because of the low energy of THz waves and the overlap of their vibration and rotational levels with biological macromolecules, the non-thermal biological effects of THz radiation may demonstrate unique features and application prospects [3].

To study the thermal effects of THz waves, Wilmink *et al*. [4] have exposed human cells to THz radiation (2.52 THz, 227 mW/cm^2^, durations of 1 ~ 40 min). They found that the vast majority of cells underwent apoptosis or necrosis after being exposed for 20 min or longer; 60% of Jurkat cells survived for 30 min, and only 20 percent lived to 40 min. In a subsequent study, human dermal fibroblasts were exposed to continuous THz radiation (2.52 THz, 84.8 mW/cm^2^, durations of 5, 10, 20, 40, and 80 minutes). It was found that cell viability was significantly affected when cellular temperatures increased by 3 °C, and the expression of both heat shock proteins and DNA damage markers tended to increase. Compared with the radiation groups, the groups subjected to hyperthermic exposure (3°C higher than physiological conditions) exhibited equivalent levels of heat shock protein expression [5]. These findings suggested that radiation at 2.52 THz generates predominantly thermal effects in mammalian cells.

As a major aspect of the study of related mechanisms, the non-thermal biological effects of THz waves have received greater attention. Bourne *et al*. [6] have exposed primarily cultured human keratinocytes and ND 7/23 cell lines characterized by sensory neurons to 0.14 THz radiation for 80 ns, with power densities ranging from 24 mW/cm^2^ to 62 mW/cm^2^, but no changes occurred in the glutathione and heat shock protein 70 levels, which are typically indicators of the degree of stress response. Through the observation of the effects of THz radiation on neurons *in vitro*, Ol’shevskaia *et al*. [7] have observed that THz radiation causes injury to the morphology of neurons in a power- and wavelength-dependent manner.

Meanwhile, there have been many negative results in the study of the biological effects of THz radiation. Studies have indicated no effects of THz radiation on the differentiation or cell viability of human keratinocytes or neurons at a frequency of 0.14 THz or a power of 0.45 J/cm^2^ [6,8]. In addition, THz radiation has also been found to have no significant effect on the morphology, adhesion, proliferation and differentiation of human epithelial and embryonic stem cells as well as the chromosomes of human lymphocytes [9,10].

## Biological mechanisms involved in THz radiation

THz radiation may interact with cellular components at multiple levels, including chromosomes, DNA (deoxyribonucleic acid), genes and proteins.

At the level of DNA and chromosomes, Hintzsche *et al*. [11] have exposed a monolayer-cultured human-hamster hybrid cell line to a 0.106 THz radiation source for 0.5 h, with power densities ranging from 0.043 mW/cm^2^ to 4.3 mW/cm^2^. The results indicated that the THz radiation affected chromatid separation during the mitotic anaphase and telophase. Recently, several studies have indicated that the non-thermal effects of THz radiation may affect the stability of DNA by establishing a system *in vitro*, leading to chromosomal aberrations of human lymphocytes and genetic changes during the differentiation of mouse stem cells [12-16].

At the levels of genes and protein, the USA Air Force Research Laboratory [5] has studied the genetic changes in Jurkat cells exposed to continuous THz radiation (2.52 THz, 227 mW/cm^2^, durations of 1 ~ 40 min). They observed that the THz radiation primarily affected genes encoding inflammatory cytokines, including IL2-inducible T-cell kinase (ITK), integrin-linked kinase-associated serine/threonine phosphatase (ILRAP), RAD1, interleukin 6 (IL6), and interleukin 8 (IL8). Bock *et al*. [13] have investigated the relationship between broad-spectrum THz radiation and genetic changes and have found that the application of THz irradiation accelerated cell differentiation by activating the transcription factor peroxisome proliferator-activated receptor gamma (PPARγ), indicating that THz radiation may be a potential tool for cellular reprogramming. Alexandrov *et al*. [14,15] have observed changes in gene expression in mesenchymal stem cells following exposure to 2.52 THz radiation for 2 h under non-thermal conditions, including changes in adiponectin; solute carrier family 2 (facilitated glucose transporter), member 4 (GLUT4); and PPARγ. Titova *et al*. [17,18] have exposed human skin tissue to 0.2 ~ 2.5 THz radiation for 10 min. As a result, the expression of more than half of the epidermal differentiation complex (EDC), which is predominantly involved in epidermal differentiation and is often highly expressed in skin cancer and other diseases, changed at the gene level. At the same time, they also observed that THz radiation could induce significant alterations in the phosphorylation of H2AX, indicating that THz radiation could lead to potential DNA damage in skin tissues. Kim *et al*. [19] have irradiated mouse skin with femtosecond-terahertz (fs-THz) radiation and, through genomic analysis, found that transforming growth factor-beta (TGF-β) mediated the damage response of the skin. Recently, E. coli/pKatG-gfp biosensor cells have been used to study the biological effects of THz radiation. The results demonstrated that exposure to THz radiation at wavelengths of 130, 150, and 200 μm and a power of 1.4 W/cm^2^ induced changes in green fluorescent protein (GFP) fluorescence values and thus modified the expression of GFP [20].

However, there have also been some negative results in the study of the biological mechanisms involved in THz radiation. Hintzsche *et al*. [21] have exposed HaCaT and HDF cells to THz radiation for 2 h, 8 h, and 24 h at various power intensities ranging from 0.04 to 2 mW/cm^2^, and no DNA strand breakage or chromosomal damage was observed. In their follow-up study, at higher frequencies (0.380 and 2.520 THz), non-ionizing radiation did not induce genomic damage in human skin cells [22].

## Research on materials for protection against THz radiation

The development and application of protective/shielding materials constitute an effective means of ensuring the survival of personnel and weapon systems for future battlefield applications of technologies; such studies are therefore highly valued in the world’s major military nations. Many patents have been filed abroad in regard to protection/shielding materials that are compatible with multiple bands, and several domestic units have also developed multi-band camouflage screens that are compatible with visible light, infrared, and radar and are designed for stealth to achieve multi-band protection/shielding. Although considerable achievements have been made with respect to protection/shielding materials that are effective against multiple bands, including the microwave and infrared bands, a great deal of development is still required in the THz band.

Liu *et al*. [23] have studied the THz spectra of four stealth cloths, including gray shielding cloth, brick red shielding cloth, and light and dark calibration shielding cloth. The results indicated that the shielding effectiveness of these four cloths was no less than 30 dB, suggesting that these cloths could be effective in shielding electronic devices against microwave or THz waves to prevent information leakage or interference. The gray and brick red shielding cloths exhibited better performance, with shielding effectiveness exceeding 60 dB and as high as 72.5 dB in the band from 0.33 to 1.15 THz. These four shielding cloths demonstrated a good shielding effect against THz radiation, especially the gray shielding cloth. These cloths would considerably hinder THz detection if they were to be applied to aerospace or military equipment.

Thus far, however, most studies have focused on the shielding effects of materials for electronic equipment; there has been very little investigation into the protection of humans against THz radiation. Therefore, one of the key problems currently facing the field of THz research is the study of materials for the protection of humans from THz-wave radiation.

## Conclusions

As a typical frontier cross-disciplinary science, THz science integrates the characteristics of both electronics and photonics and represents various unique mechanisms and methods that require elucidation, with the potential to lay the foundation for the next generation of science and technology and promote the development of information technology after the Moore era. THz science may lead to revolutionary developments and breakthroughs in science and technology, including military applications. However, studies of the biological effects of THz radiation are still lacking, far behind the development of the corresponding applied research. Therefore, cross-integration involving physics, biology, medicine, materials science and other fields should be strengthened in the field of THz science to advance the research and biomedical applications of THz waves. The scientific problems that have yet to be resolved include the interaction between THz waves and biological macromolecules, the mechanisms of this interaction, the biological effects of THz radiation on specific tissues and cells, and the key technologies and equipment involved in the biomedical applications of THz radiation.

The emergence and development of each new major technology for biological spectroscopy has caused a revolution in the field of biology. As an important strategic resource, THz science will contribute immeasurably to the life sciences and clinical medicine. However, the determination of whether it can play a role as significant as that of X-rays still requires a global effort.

## Abbreviations

THz: Terahertz
GHz: Giga Hz
DNA: Deoxyribonucleic acid
ITK: IL2 inducible T cell kinase
ILRAP: Integrin-linked kinase-associated serine/threonine phosphatase
IL6: Interleukin 6
IL8: Interleukin 8
PPARγ: Peroxisome proliferator-activated receptor gamma
GLUT4: Solute carrier family 2 (facilitated glucose transporter), member 4
EDC: Epidermal differentiation complex
fs-THz: Femtosecond-terahertz
TGF-β: Transforming growth factor-beta
GFP: Green fluorescent protein
